# Adherence to an antioxidant diet and lifestyle is associated with reduced risk of cardiovascular disease and mortality among adults with nonalcoholic fatty liver disease: evidence from NHANES 1999–2018

**DOI:** 10.3389/fnut.2024.1361567

**Published:** 2024-04-08

**Authors:** Yingzi Li, Yipin Liu

**Affiliations:** Department of Gastroenterology, Yantai Affiliated Hospital of Binzhou Medical University, Yantai, Shandong, China

**Keywords:** NAFLD, antioxidant, CVD, mortality, oxidative stress

## Abstract

**Background:**

Nonalcoholic fatty liver disease (NAFLD) stands a prevalent chronic liver condition significantly influenced by oxidative stress. We investigated the unclear relationship between antioxidant-rich diet and lifestyle and cardiovascular disease (CVD) prevalence rate and mortality in adult patients with NAFLD.

**Methods:**

This study utilized data from the National Health and Nutrition Examination Survey (NHAENS) spanning from 1999 to 2018 to investigate the association between adherence to an antioxidant-rich diet and lifestyle and the cardiovascular disease (CVD) prevalence rate and mortality in adult patients with NAFLD. The study employed the Oxidative Balance Score (OBS) to define antioxidant diet and lifestyle.

**Results:**

Including 8,670 adult patients with NAFLD, the study revealed an inverse association between OBS and the prevalence of most CVD conditions. Fully adjusted models demonstrated that each unit increase in diet OBS, lifestyle OBS, and overall OBS corresponded to a 2, 7, and 2% reduction in all-cause mortality, respectively. In models 2, findings revealed that lifestyle Q2 and Q3 were linked to reduced cancer mortality, whereas diet and overall OBS did not exhibit an association. Additionally, Stratified analysis revealed that age (<45 years) and education level (> high school) significantly influenced the association between the OBS and the prevalence of CVD.

**Conclusion:**

These results underscore the protective link between adherence to an antioxidant diet and lifestyle and a diminished prevalence of CVD and mortality in adults with NAFLD, particularly among younger and higher-educated populations.

## 1 Introduction

Nonalcoholic fatty liver disease (NAFLD) stands as the predominant cause of chronic liver disease on a global scale, affecting approximately 25% of the world’s population (1, 2). Manifesting in a spectrum of pathological features, NAFLD ranges from simple intrahepatic lipid accumulation, characterized by steatosis and non-alcoholic fatty liver (NAFL), to the progression of non-alcoholic steatohepatitis (NASH), encompassing varying degrees of necrotizing inflammation, accelerated fibrosis, cirrhosis, and ultimately hepatocellular carcinoma (HCC) (3, 4). Driven by shifts in dietary patterns, lifestyle choices, and an aging population, the incidence of NAFLD is on the rise, emerging as a pervasive global health concern (5). Beyond its impact on liver function, research has elucidated a significant association between NAFLD and cardiovascular disease (CVD). Notably, NAFLD has been identified as an independent risk factor for the onset of CVD, a leading contributor to global of morbidity and mortality (6, 7).

Currently, no pharmaceuticals are specifically endorsed for the treatment of NAFLD, making lifestyle modification and the management of risk factors the foremost therapeutic approach for patients with NAFLD (8). Consequently, antioxidant-based dietary and lifestyle interventions have attracted attention as potential strategies to diminish the risk of NAFLD progression and related complications. Despite an incomplete understanding of NAFLD’s pathogenesis, numerous studies have emphasized the pivotal role of oxidative stress, stemming from an imbalance in oxidative and antioxidant processes, in the initiation and progression of NAFLD (9–11). Dietary and lifestyle adjustments rich in antioxidants, such as regular exercise and smoking cessation, have been proposed (12). Research (13–15) has indicated that various antioxidants, including vitamin E and polyphenolic compounds, can ameliorate NAFLD/NASH by inhibiting lipid oxidation and accumulation. These interventions aim to modulate reactive oxygen species, consequently alleviating oxidative stress, improving liver function, and reducing liver-related morbidity and mortality.

While numerous studies have explored the impact of antioxidant diets and lifestyles on liver-related outcomes, the relationship between these interventions and the prevalence of CVD and mortality in adult NAFLD patients remains understudied. Thus, our investigation was based on data from the US National Health and Nutrition Examination Survey (NHANES) combined with the OBS assessment tool to understand the association and potential benefits of antioxidant diets and lifestyles in the prevalence of CVD and mortality among NAFLD patients. We aim to mitigate the disease burden and improve outcomes in this population.

## 2 Methods

### 2.1 Study design and population

NHANES is a research program strategically devised to evaluate the health and nutritional status of non-institutionalized adult and child citizens in the United States, representing a pivotal initiative of the National Center for Health Statistics (NCHS). Commencing in the early 1960s, the NHANES program has evolved, and since 1999, it has been an ongoing survey conducted on a biennial cycle. The NHANES survey encompasses interviews and physical examinations. The household interviews incorporate diverse questionnaires, covering demographic, socioeconomic, nutritional, and health-related aspects. The physical examinations include anthropometric measurements, and laboratory tests, among others, conducted at the Mobile Examination Center. Consequently, NHANES constitutes a continuous, nationally representative, population-based series of cross-sectional surveys distinguished by intricate designs and probability sampling.

All NHANES survey cycles underwent rigorous review approval by the NCHS Ethics Review Board (ERB), and written consent was secured from all participants (please refer to https://www.cdc.gov/nchs/nhanes/irba98.htm for the ethical review and consent protocols for each cycle). NHANES is a publicly accessible database, enabling researchers to freely download data and related resources for conducting pertinent research. Given that the data were previously collected and made publicly available by the Center for Disease Control and Prevention, no additional ethical review consent was necessary for this study.

**Figure 1:**
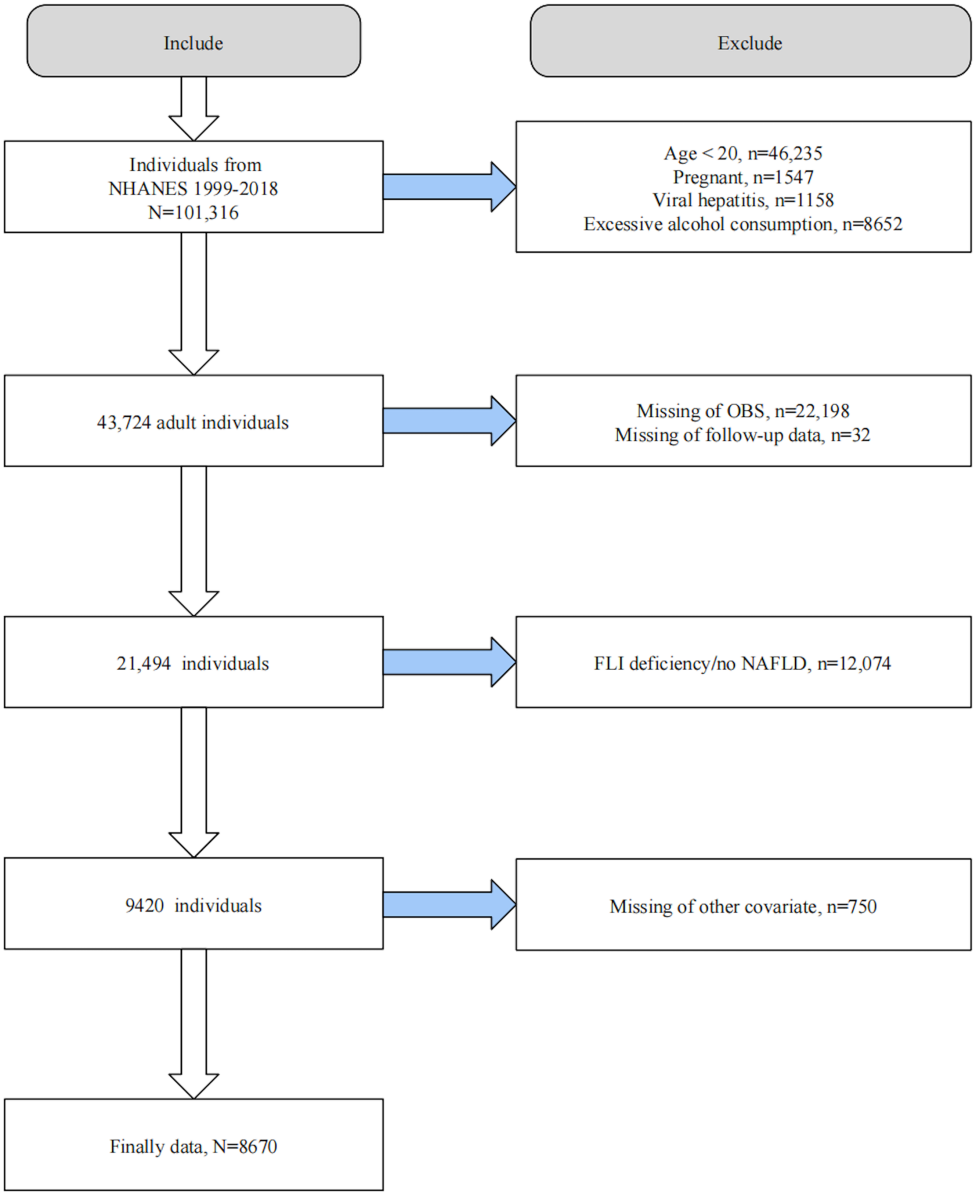
Flow chart of participant selection. OBS, Oxidative balance score; NAFLD, Nonalcoholic fatty liver disease; FLI, Fatty liver index; NHAENS, National Health and Nutrition Examination Survey.

We initially screened 101,316 participants from NHANES 1999–2018 excluding those underage (*n* = 46,235), pregnant (*n* = 1,547), with viral hepatitis (*n* = 1,158), and consumed consuming excessive alcohol (i.e., ≥3 drinks/d in males and ≥ 2 drinks/d in females; *n* = 8,652). Subsequently, we excluded 22,198 participants lacking information on OBS, 32 with missing follow-up data, 12,074 without FLI diagnostic information, and 750 lacking covariates information. Ultimately, 8,670 participants were included for further analysis (Figure 1).

### 2.2 Definition of OBS

Numerous studies have previously demonstrated the viability of OBS using the NHANES database. We defined the OBS utilizing well-validated components and calculation schemes derived from prior NHANES investigations (11, 16). The OBS comprises the dietary OBS, consisting of 16 dietary components, and the lifestyle OBS, comprising 4 lifestyle components. Dietary intake assessments from 1999 through 2002 were based on single 24-h face-to-face dietary recall. Since 2003–2004, NHANES has included a second 24-h dietary recall, conducted by telephone interview approximately 3–10 days after the initial recall (17). Thus, for the period 1999–2002, we utilized data from a single dietary intake recall interview, while from 2003 onwards, we used the average of the two dietary recalls as intake. Evaluation of dietary nutrient intake was based on the USDA Nutrient Survey database (18). Nutrient intake from dietary supplements and medications was not included due to insufficient detail in earlier NHANES cycles (19).

Physical activity was quantified in metabolic equivalents (MET) using the Physical activity questionnaire (20). Serum cotinine, a metabolite of nicotine, served as a measure of smoking, providing more accurate assessment of both active smoking and exposure to secondhand smoke (21).

We assigned gender-specific scores to all OBS components. Antioxidant components were scored of 0, 1, and 2 based on tertiles, with 0 for the lowest and 2 for the highest tertile, while the scoring was reversed for pro-oxidants.

For alcohol consumption, we assigned scores of 0, 1, and 2 to the >3, 2–3, and ≤ 2 drinks/day distributions of alcohol consumption in the past year for men, and scores of 0, 1, and 2 to the >2, 1–2, and ≤ 1 drinks/day for women. The scoring scheme of OBS is summarized in Supplementary Table S1.

### 2.3 Definition of NAFLD

We utilized the well-established FLI as a surrogate marker for NAFLD, calculated from serum triglycerides (TG), body mass index (BMI), waist circumference (WC), and serum gamma-glutamyl transpeptidase (GGT) using the formula: FLI = (e^0.953*loge (TG) + 0.139*BMI + 0.718*loge (GGT) + 0.053*WC − 15.745^) / (1 + e^0.953*loge (TG) + 0.139*BMI + 0.718*loge (GGT) + 0.053*WC − 15.745^) × 100 (22). FLI demonstrates good accuracy in detecting hepatic steatosis, with an area under the curve of 0.84. Suspected NAFLD was defined as FLI ≥ 60 after excluding other chronic liver diseases, such as excessive alcohol consumption and viral hepatitis. Additionally, we conducted a sensitivity analysis using another widely used noninvasive diagnostic method for NAFLD, the US Fatty Liver Index (USFLI). The formula of USFLI was USFLI = (e^−0.8073 × non-Hispanic black + 0.3458 × Mexican American + 0.0093 × age + 0.6151 × ln (GGT) + 0.0249 × WC + 1.1792 × ln (insulin) + 0.8242 × ln (glucose) − 14.7812^)/(1 + e ^−0.8073 × non-Hispanic black + 0.3458 × Mexican American + 0.0093 × age + 0.6151 × ln (GGT) + 0.0249 × WC + 1.1792 × ln (insulin) + 0.8242 × ln (glucose) − 14.7812^) × 100 (23). A USFLI ≥30 was considered indicative of NAFLD.

### 2.4 Determination of outcome variables

The outcomes assessed in our study were the prevalence of CVD and all-cause, as well as cause-specific mortality among patients with NAFLD. The presence of any diagnosed CVD, encompassing coronary heart disease (CHD), stroke, heart attack, congestive heart failure (CHF), and angina pectoris, was determined based on self-reporting from the NHANES questionnaire. Mortality information was obtained through a prospective matching of the NHANES 1999–2018 baseline population with the National Death Index database, with follow-up data available until December 1, 2019. Mortality data included all-cause mortality, CVD mortality, and cancer-related mortality. CVD mortality was identified using ICD-10 codes I00-I09, I11, I13, I20-I51, or I60-I69, while cancer-related mortality was determined by ICD-10 codes C00-C97.

### 2.5 Covariates

We selected several crucial potential covariates based on previous research. These covariates included age, gender, ethnicity (Mexican American, non-Hispanic black, non-Hispanic white, other Hispanic, or other races), education level (<high school, high school, or > high school), marital status (single or non-single), family income to poverty (PIR), dietary total energy intake, diabetes, and hypertension. Diabetes was diagnosed based on self-reports, a HbA1c > 6.5%, fasting blood glucose >7.0 mmoL/L, a 2-h oral glucose tolerance test blood glucose ≥11.1 mmoL/L, or the use of anti-diabetic medication. Hypertension was diagnosed by self-reported hypertension, blood pressure ≥ 140/90 mmHg, or the use of anti-hypertensive drugs. When investigating the association of OBS with mortality in NAFLD, we additionally adjusted for baseline CVD status.

### 2.6 Statistical analysis

All analyses were conducted using EmpowerStats (X&Y Solutions, Inc., Boston, MA) and R software version 4.2.3 (The R Foundation). We applied weights according to the NHANES Analytic Guidelines to ensure the sample’s representative of the entire U.S. noninstitutionalized population. Continuous variables were presented as mean ± standard error, and categorical variables were reported as numbers (percentages) in the baseline analysis. One-way ANOVA was employed for analyzing continuous variables, while chi-square tests were utilized for categorical variables.

Firstly, we examined the relationship between OBS and CVD prevalence in NAFLD using multivariate-adjusted logistic regression analysis. For assessing the association of OBS with mortality in NAFLD, multivariate-adjusted Cox regression analyses were employed to ascertain the independent prognostic value of OBS in NAFLD. We established three multivariate-adjusted models in logistic and Cox regressions. The crude model (Model 0) had no covariate adjustments. Model 1 partially adjusted for age, sex, race, education, PIR, and marital status. Model 2 additionally incorporated total dietary energy intake, diabetes, and hypertension (and additionally adjusted for baseline CVD status in Cox regression analysis) in addition to the adjustments made in Model 1.

We employed restricted cubic spline (RCS) modeling and smoothed curve fitting to investigate potential nonlinear relationships. The curve-fitting term was defined by the RCS function in the rms package. To assess the consistency of these relationships across subgroups, we conducted stratified and interaction analyses to identify potential effect modifiers. Lastly, we applied sensitivity analyses to test the robustness of the findings. A two-sided *p* < 0.05 was considered statistically significant in all analyses.

## 3 Results

### 3.1 Baseline characteristics

Of the 8,670 eligible NAFLD participants, the mean age was 50.59 years and 58.02% were male. In baseline analyses, higher OBS was associated with higher PIR, total energy intake, and was more likely to be non-Hispanic white, non-single, higher than high school educated, and without hypertension. Of note, higher OBS quartiles were associated with lower CVD prevalence, including all included CVD types (Table 1).

**Table 1:** Baseline characteristics according to OBS quartiles in NAFLD patients.

	Total	Q1	Q2	Q3	Q4	*P*-value
Age, year	50.59 ± 0.26	50.19 ± 0.38	51.16 ± 0.44	50.90 ± 0.45	50.24 ± 0.39	0.19
PIR	3.22 ± 0.04	2.76 ± 0.05	3.14 ± 0.05	3.29 ± 0.05	3.53 ± 0.05	<0.0001
Energy, kcal/day	2166.55 ± 12.00	1479.72 ± 17.37	1916.74 ± 16.23	2203.15 ± 16.60	2746.43 ± 21.63	<0.0001
OBS.dietary	16.63 ± 0.12	7.24 ± 0.07	13.13 ± 0.04	18.03 ± 0.04	23.95 ± 0.06	<0.0001
OBS.lifestyle	4.59 ± 0.02	4.28 ± 0.04	4.53 ± 0.04	4.64 ± 0.04	4.78 ± 0.04	<0.0001
OBS	21.22 ± 0.13	11.52 ± 0.08	17.67 ± 0.06	22.67 ± 0.06	28.73 ± 0.07	<0.0001
Sex						0.52
Male	4877(58.02)	1262(58.13)	1140(59.12)	1104(56.27)	1371(58.49)	
Female	3793(41.98)	866(41.87)	875(40.88)	943(43.73)	1109(41.51)	
Race						<0.0001
Mexican American	1419(6.55)	319(6.25)	336(7.07)	336(6.52)	428(6.40)	
Non-Hispanic Black	1738(9.73)	602(15.81)	424(10.39)	358(8.59)	354(6.25)	
Non-Hispanic White	4368(74.71)	952(68.49)	991(73.75)	1075(75.65)	1350(78.64)	
Other Hispanic	606(4.27)	147(4.67)	148(4.76)	128(3.68)	183(4.11)	
Other race	539(4.75)	108(4.78)	116(4.03)	150(5.55)	165(4.61)	
Marital status						0.003
Non-single	5789(71.62)	1338(68.02)	1329(70.65)	1394(72.27)	1728(74.09)	
Single	2881(28.38)	790(31.98)	686(29.35)	653(27.73)	752(25.91)	
Education						<0.0001
<High school	766(3.78)	273(5.85)	191(4.53)	152(3.00)	150(2.52)	
High school	3109(33.51)	921(44.37)	738(33.35)	699(31.89)	751(27.89)	
>High school	4795(62.71)	934(49.78)	1086(62.12)	1196(65.11)	1579(69.59)	
Diabetes						0.17
No	6527(80.39)	1533(78.75)	1516(80.34)	1538(79.71)	1940(81.97)	
Yes	2143(19.61)	595(21.25)	499(19.66)	509(20.29)	540(18.03)	
Hypertension						0.04
No	4003(50.07)	900(47.76)	933(49.04)	933(49.18)	1237(52.88)	
Yes	4667(49.93)	1228(52.24)	1082(50.96)	1114(50.82)	1243(47.12)	
CVD						<0.0001
No	7547(89.34)	1771(85.70)	1743(88.26)	1800(90.34)	2233(91.67)	
Yes	1123(10.66)	357(14.30)	272(11.74)	247(9.66)	247(8.33)	
Congestive heart failure						<0.0001
No	8349(97.24)	2010(95.94)	1926(96.62)	1989(97.69)	2424(98.48)	
Yes	303(2.65)	113(4.06)	86(3.38)	55(2.31)	49(1.52)	
Coronary heart disease						0.002
No	8137(94.70)	1960(93.26)	1884(94.55)	1930(95.80)	2363(95.94)	
Yes	493(4.94)	158(6.74)	118(5.45)	104(4.20)	113(4.06)	
Heart attack						0.01
No	8196(95.42)	1971(94.42)	1894(94.50)	1957(96.11)	2374(96.42)	
Yes	466(4.49)	154(5.58)	118(5.50)	89(3.89)	105(3.58)	
Stroke						0.01
No	8339(97.22)	2019(96.36)	1939(96.97)	1970(97.61)	2411(98.00)	
Yes	320(2.66)	105(3.64)	75(3.03)	74(2.39)	66(2.00)	
Angina						0.002
No	8319(96.61)	2018(95.14)	1924(96.68)	1975(97.45)	2402(97.40)	
Yes	330(3.21)	105(4.86)	85(3.32)	69(2.55)	71(2.60)	

OBS, Oxidative balance score; PIR, family income to poverty ratio; CVD, cardiovascular disease. Continuous variables were presented as mean ± standard error, and categorical variables were reported as numbers (percentages).

### 3.2 Association of OBS with prevalence of CVD in NAFLD

We used multivariate adjusted logistic regression analyses to explore the relationship between OBS and the likelihood of CVD development in the NAFLD population. In the model 2 that adjusted for all covariates, dietary OBS, lifestyle OBS, and overall OBS were all associated with lower odds of CVD [dietary OBS: odds ratio (OR) and 95% confidence interval (CI) = 0.98 (0.96, 1.00), *p* = 0.0317; lifestyle OBS: OR and 95% CI = 0.84 (0.79, 0.90), *p* < 0.0001; overall OBS: OR and 95% CI = 0.97 (0.95, 0.99), *p* = 0.0015]. As categorical variables, dietary OBS in Q3 and Q4 (compared to Q1) was associated with 27 and 25% reductions in the likelihood of CVD, respectively, whereas lifestyle OBS in Q3 and Q4 was associated with 33 and 45% reductions in odds, respectively. In addition, overall OBS at Q2, Q3, and Q4 was associated with 29, 39, and 40% reductions in the likelihood of CVD. A potential dose–response relationship associated with reduced prevalence of CVD was observed in all OBS (Table 2). Multivariable-adjusted regression analyses of the association between OBS and specific types of CVD showed that OBS was inversely associated with the prevalence of most CVDs, except stroke. A particularly intriguing detail emerged regarding congestive heart failure and heart attacks. While not significantly associations were found between these conditions and either dietary or overall OBS, a discernible trend of risk reduction was observed in the Q4 grouping of Model 1 for lifestyle OBS (Supplementary Table S2).

**Table 2:** The relationship between OBS and the likelihood of CVD development in the NAFLD population.

Outcome: CVD	Crude model OR (95%CI) *P*-value	Model 1 OR (95%CI) *P*-value	Model 2 OR (95%CI) *P*-value
OBS.DIETARY	0.96 (0.95, 0.98) <0.0001	0.98 (0.96, 0.99) 0.0030	0.98 (0.96, 1.00) 0.0317
OBS.DIETARY			
Q1	Ref.	Ref.	Ref.
Q2	0.80 (0.66, 0.97) 0.0215	0.83 (0.67, 1.04) 0.1139	0.85 (0.67, 1.08) 0.1933
Q3	0.64 (0.50, 0.83) 0.0008	0.70 (0.54, 0.93) 0.0133	0.73 (0.55, 0.98) 0.0360
Q4	0.54 (0.44, 0.68) <0.0001	0.68 (0.53, 0.88) 0.0041	0.75 (0.55, 1.01) 0.0587
*P* for trend	<0.0001	0.0034	0.0428
OBS.LIFESTYLE	0.95 (0.89, 1.00) 0.0745	0.83 (0.78, 0.89) <0.0001	0.84 (0.79, 0.90) <0.0001
OBS.LIFESTYLE			
Q1	Ref.	Ref.	Ref.
Q2	1.14 (0.96, 1.34) 0.1300	0.97 (0.80, 1.17) 0.7445	0.98 (0.81, 1.20) 0.8760
Q3	0.88 (0.71, 1.09) 0.2444	0.66 (0.52, 0.83) 0.0008	0.67 (0.53, 0.85) 0.0013
Q4	0.84 (0.67, 1.06) 0.1478	0.54 (0.42, 0.69) <0.0001	0.55 (0.43, 0.71) <0.0001
*P* for trend	0.0328	<0.0001	<0.0001
OBS	0.97 (0.95, 0.98) <0.0001	0.97 (0.96, 0.99) 0.0003	0.97 (0.95, 0.99) 0.0015
OBS			
Q1	Ref.	Ref.	Ref.
Q2	0.70 (0.58, 0.85) 0.0004	0.72 (0.57, 0.91) 0.0064	0.71 (0.55, 0.91) 0.0088
Q3	0.57 (0.44, 0.73) <0.0001	0.61 (0.46, 0.81) 0.0008	0.61 (0.45, 0.82) 0.0015
Q4	0.52 (0.41, 0.65) <0.0001	0.59 (0.45, 0.78) 0.0003	0.60 (0.43, 0.83) 0.0027
*P* for trend	<0.0001	0.0004	0.0030

Model 1, age, sex, race, marital, education, PIR; Model 2, Model1+diabetes+hypertension+energy intake. OBS, Oxidative balance score; NAFLD, Nonalcoholic fatty liver disease; CVD, cardiovascular disease; PIR, family income to poverty ratio; OR, odds ratio; 95% CI, 95% confidence interval; ref, reference.

### 3.3 Association of OBS with mortality in patients with NAFLD

After a median follow-up of 115 months (interquartile range = 62.2–168.0 months), 1,246 (10.56%) patients with NAFLD died, with 421 and 322 CVD- and cancer-related deaths, respectively (Supplementary Table S3). In the fully adjusted model, each unit increase in dietary OBS, lifestyle OBS, and overall OBS was associated with a 2, 7, and 2% reduction in all-cause mortality, respectively (dietary OBS: HR = 0.98, *p* = 0.003; lifestyle OBS: HR = 0.93, *p* = 0.01; and overall OBS: HR = 0.98, *p* = 0.001), and all had a potential dose–response relationship. Dietary OBS and overall OBS, but not lifestyle OBS, were associated with reduced CVD mortality (dietary OBS: HR = 0.97, *p* = 0.01; lifestyle OBS: HR = 0.93, *p* = 0.11; overall OBS: HR = 0.96, *p* = 0.01). However, we only observed that lifestyle OBS at Q2 and Q3 were associated with reduced cancer mortality, while none of the other OBS were associated with cancer mortality (Tables 3–5).

**Table 3:** The relationship between OBS and all-cause mortality in patients with NAFLD.

All-cause	Crude model HR (95%CI) *P*-value	Model 1 HR (95%CI) *P*-value	Model 2 HR (95%CI) *P*-value
OBS.DIETARY	0.96(0.95, 0.97) <0.0001	0.98(0.96, 0.99) <0.0001	0.98(0.96, 0.99) 0.003
OBS.DIETARY			
Q1	Ref	Ref	Ref
Q2	0.78(0.64, 0.94) 0.01	0.82(0.68, 0.98) 0.03	0.85(0.70, 1.03) 0.1
Q3	0.73(0.62, 0.86) <0.001	0.80(0.68, 0.95) 0.01	0.85(0.70, 1.02) 0.08
Q4	0.46(0.38, 0.56) <0.0001	0.64(0.53, 0.77) <0.0001	0.69(0.54, 0.88) 0.003
*P* for trend	<0.0001	<0.0001	0.005
OBS.LIFESTYLE	1.07(1.01, 1.14) 0.02	0.92(0.87, 0.97) 0.003	0.93(0.88, 0.99) 0.01
OBS.LIFESTYLE			
Q1	Ref	Ref	Ref
Q2	1.11(0.91, 1.35) 0.3	0.89(0.72, 1.10) 0.28	0.91(0.74, 1.11) 0.35
Q3	0.92(0.72, 1.17) 0.49	0.63(0.50, 0.78) <0.0001	0.65(0.52, 0.81) <0.001
Q4	1.32(1.07, 1.62) 0.01	0.78(0.64, 0.95) 0.01	0.82(0.67, 1.00) 0.05
*P* for trend	0.08	<0.001	0.005
OBS	0.97(0.96, 0.98) <0.0001	0.97(0.96, 0.98) <0.0001	0.98(0.96, 0.99) 0.001
OBS			
Q1	Ref	Ref	Ref
Q2	0.85(0.71, 1.00) 0.05	0.88(0.75, 1.04) 0.13	0.91(0.77, 1.09) 0.32
Q3	0.62(0.51, 0.75) <0.0001	0.68(0.57, 0.82) <0.0001	0.72(0.58, 0.89) 0.002
Q4	0.52(0.43, 0.64) <0.0001	0.66(0.54, 0.80) <0.0001	0.71(0.55, 0.92) 0.01
*P* for trend	<0.0001	<0.0001	0.003

Model 1, age, sex, race, marital, education, PIR; Model 2, Model1+diabetes+hypertension+CVD+energy intake. OBS, Oxidative balance score; NAFLD, Nonalcoholic fatty liver disease; CVD, cardiovascular disease; PIR, family income to poverty ratio; HR, hazard ratio; 95% CI, 95% confidence interval; ref, residuals from fitted values.

**Table 4:** The relationship between OBS and CVD-cause mortality in patients with NAFLD.

CVD-cause	Crude model HR (95%CI) *P*-value	Model 1 HR (95%CI) *P*-value	Model 2 HR (95%CI) *P*-value
OBS.DIETARY	0.95(0.93, 0.97) <0.0001	0.97(0.95, 0.99) 0.001	0.97(0.94, 0.99) 0.01
OBS.DIETARY			
Q1	Ref	Ref	Ref
Q2	0.82(0.58, 1.14) 0.23	0.85(0.62, 1.16) 0.31	0.88(0.66, 1.18) 0.38
Q3	0.68(0.49, 0.95) 0.02	0.75(0.55, 1.03) 0.08	0.76(0.54, 1.08) 0.12
Q4	0.37(0.25, 0.55) <0.0001	0.53(0.34, 0.80) 0.003	0.54(0.32, 0.90) 0.02
*P* for trend	<0.0001	0.002	0.02
OBS.LIFESTYLE	1.08(0.98, 1.20) 0.1	0.91(0.83, 1.00) 0.05	0.93(0.85, 1.02) 0.11
OBS.LIFESTYLE			
Q1	Ref	Ref	Ref
Q2	1.18(0.82, 1.69) 0.37	0.91(0.62, 1.33) 0.64	0.95(0.66, 1.36) 0.77
Q3	1.04(0.71, 1.53) 0.84	0.67(0.47, 0.97) 0.03	0.71(0.50, 1.00) 0.05
Q4	1.36(0.93, 1.99) 0.12	0.75(0.51, 1.09) 0.13	0.80(0.56, 1.15) 0.23
*P* for trend	0.21	0.05	0.1
OBS	0.96(0.94, 0.97) <0.0001	0.97(0.94, 0.99) 0.001	0.96(0.94, 0.99) 0.01
OBS			
Q1	Ref	Ref	Ref
Q2	0.82(0.60, 1.13) 0.22	0.86(0.63, 1.16) 0.32	0.90(0.66, 1.21) 0.48
Q3	0.62(0.44, 0.87) 0.01	0.68(0.48, 0.94) 0.02	0.70(0.49, 1.00) 0.05
Q4	0.43(0.28, 0.67) <0.001	0.56(0.35, 0.87) 0.01	0.59(0.35, 0.98) 0.04
*P* for trend	<0.0001	0.004	0.02

Model 1, age, sex, race, marital, education, PIR; Model 2, Model1+diabetes+hypertension+energy intake. OBS, Oxidative balance score; NAFLD, Nonalcoholic fatty liver disease; CVD, cardiovascular disease; PIR, family income to poverty ratio; HR, hazard ratio; 95% CI, 95% confidence interval; ref, residuals from fitted values.

**Table 5:** The relationship between OBS and cancer-cause mortality in patients with NAFLD.

Cancer-cause	Crude model HR (95%CI) *P*-value	Model 1 HR (95%CI) *P*-value	Model 2 HR (95%CI) *P*-value
OBS.DIETARY	0.97(0.95, 0.99) 0.01	0.98(0.96, 1.01) 0.16	0.98(0.95, 1.01) 0.21
OBS.DIETARY			
Q1	Ref	Ref	Ref
Q2	0.80(0.53, 1.19) 0.27	0.80(0.53, 1.22) 0.3	0.80(0.53, 1.21) 0.3
Q3	0.89(0.64, 1.24) 0.48	0.94(0.66, 1.34) 0.74	0.94(0.64, 1.39) 0.77
Q4	0.58(0.40, 0.86) 0.01	0.75(0.50, 1.12) 0.16	0.75(0.46, 1.22) 0.25
*P* for trend	0.01	0.27	0.4
OBS.LIFESTYLE	1.08(0.95, 1.23) 0.23	0.92(0.80, 1.06) 0.26	0.93(0.80, 1.07) 0.29
OBS.LIFESTYLE			
Q1	Ref	Ref	Ref
Q2	0.76(0.57, 1.02) 0.07	0.60(0.44, 0.81) <0.001	0.60(0.44, 0.81) <0.001
Q3	0.80(0.52, 1.25) 0.34	0.54(0.35, 0.85) 0.01	0.55(0.34, 0.86) 0.01
Q4	1.24(0.84, 1.83) 0.28	0.71(0.47, 1.09) 0.12	0.72(0.46, 1.11) 0.14
*P* for trend	0.38	0.19	0.21
OBS	0.98(0.96, 1.00) 0.02	0.98(0.96, 1.00) 0.1	0.98(0.95, 1.01) 0.15
OBS			
Q1	Ref	Ref	Ref
Q2	0.90(0.62, 1.29) 0.56	0.89(0.62, 1.27) 0.51	0.86(0.60, 1.24) 0.43
Q3	0.73(0.50, 1.05) 0.09	0.75(0.51, 1.11) 0.15	0.72(0.46, 1.13) 0.15
Q4	0.62(0.41, 0.93) 0.02	0.70(0.47, 1.06) 0.09	0.66(0.39, 1.12) 0.12
*P* for trend	0.01	0.07	0.11

Model 1, age, sex, race, marital, education, PIR; Model 2, Model1+diabetes+hypertension+energy intake. OBS, Oxidative balance score; NAFLD, Nonalcoholic fatty liver disease; CVD, cardiovascular disease; PIR, family income to poverty ratio; HR, hazard ratio;; 95% CI, 95% confidence interval; ref, residuals from fitted values.

### 3.4 RCS analysis

**Figure 2:**
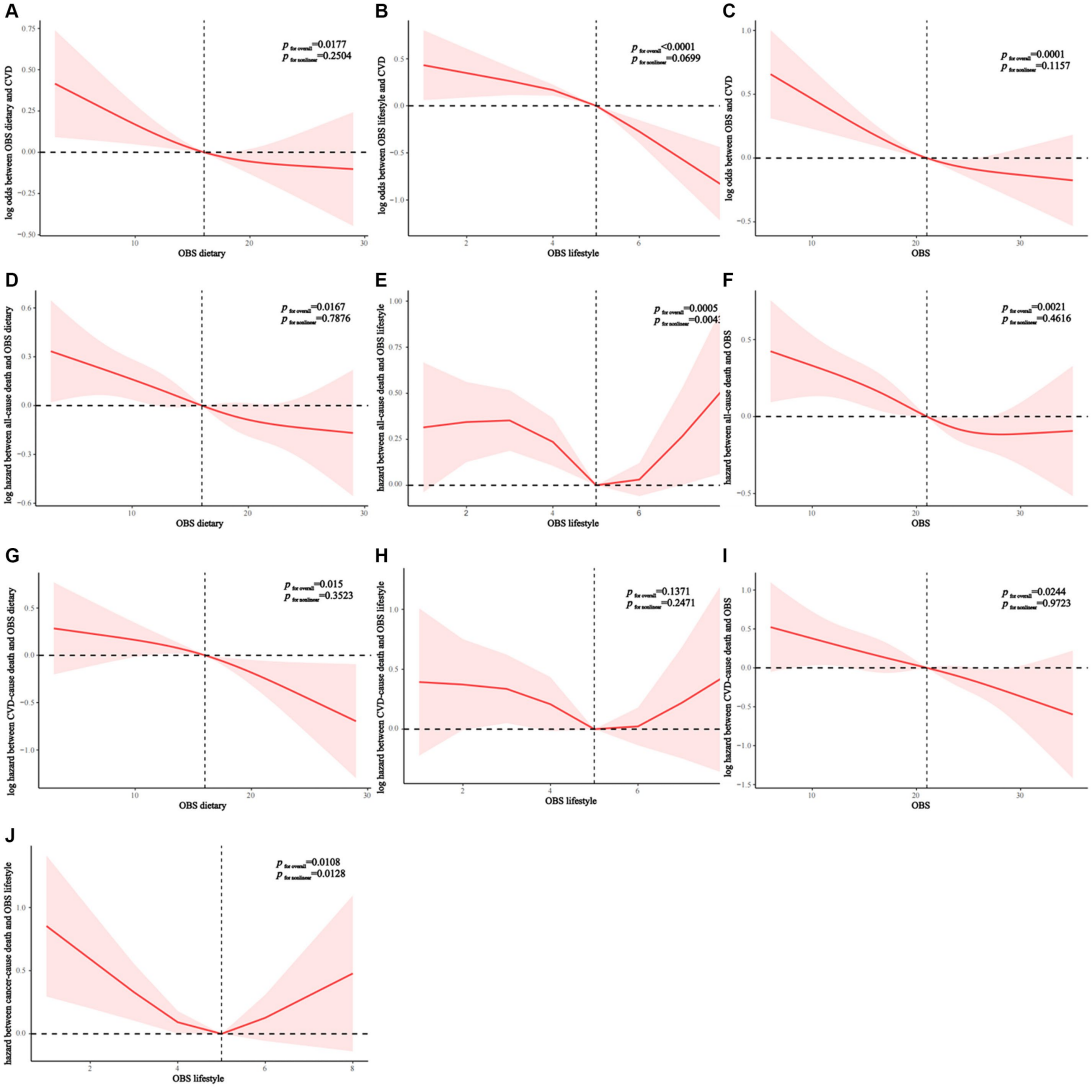
**(A–C)** OBS linear relationship with CVD morbidity rates of patients with NAFLD. **(D–F)** Diet **(D)** and lifestyle **(E)** OBS has A linear relation with NAFLD all-cause mortality. Nonlinear relationship between overall OBS **(F)** and all-cause mortality. **(G–I)** OBS linear relationship with CVD mortality rates of patients with NAFLD. **(J)** Nonlinear relationship between lifestyle OBS and cancer mortality. OBS, Oxidative balance score; NAFLD, Nonalcoholic fatty liver disease; CVD, cardiovascular disease.

RCS analysis indicated that all OBS showed a generally linear association with the prevalence of CVD in NAFLD (dietary OBS: p nonlinear = 0.2504; lifestyle OBS: p nonlinear = 0.0699; overall OBS: p nonlinear = 0.1157) (Figures 2A–C). Dietary and lifestyle OBS showed a linear association with all-cause mortality in NAFLD (dietary OBS: p nonlinear = 0.7876; lifestyle OBS: p nonlinear = 0.4616) whereas overall OBS was nonlinearly associated with all-cause mortality (p nonlinear = 0.0043) (Figures 2D–F). All OBS showed a linear association with CVD mortality in NAFLD (dietary OBS: p nonlinear = 0.3523; lifestyle OBS: p nonlinear = 0.2471; overall OBS: p nonlinear = 0.9723) (Figures 2G,H). Finally, we found that lifestyle OBS was nonlinearly associated with cancer mortality (p nonlinear = 0.0128) (Figure 2J).

### 3.5 Stratified analysis

**Figure 3:**
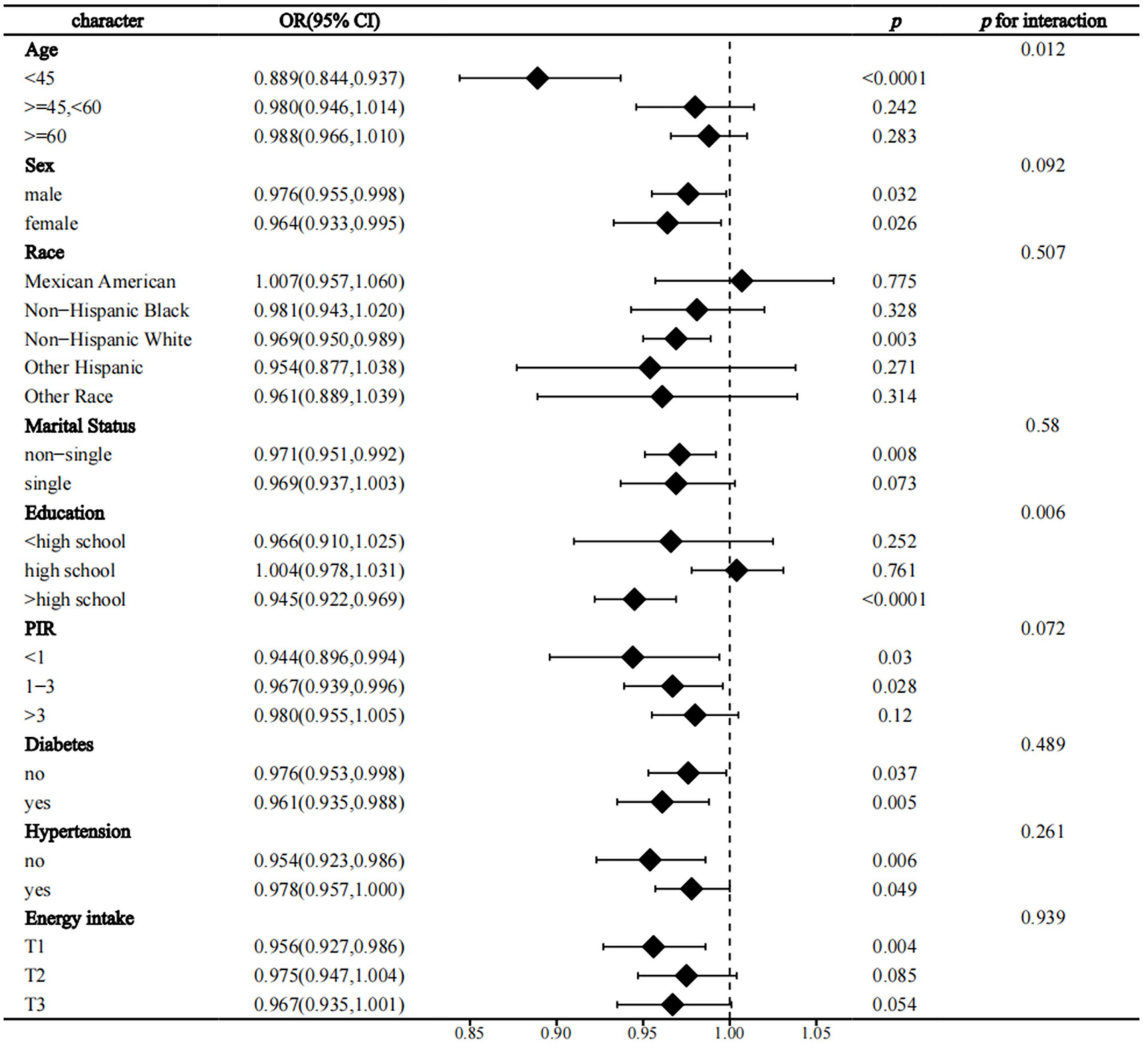
Forest plot the relationship between OBS and the prevalence of CVD in NAFLD. OBS, Oxidative balance score; NAFLD, Nonalcoholic fatty liver disease; CVD, cardiovascular disease; PIR, family income to poverty ratio; OR, odds ratio; 95% CI, 95% confidence interval.

In interaction analyses within strata, we found that age and education level were two significant effect modifiers of the relationship between OBS and the prevalence of CVD in NAFLD (p for interaction = 0.012 and 0.006, respectively). The protective effect of OBS on CVD was only present in people younger than 45 years of age and those with more than a high school education (Figure 3). Similarly, we found that education level significantly influenced the protective effect of OBS on all-cause and CVD mortality (p for interaction = 0.004 and 0.026, respectively) and that these beneficial effects were also only present in the population with education beyond high school (Supplementary Figure S1; Figure 2). Finally, we found that age was an effect modifier of the protective effect of OBS on cancer mortality (p for interaction = 0.013) (Supplementary Figure S3).

Analyses revealed significant interactions between education (>high school) and dietary OBS in relation to all-cause mortality (p for interaction<0.001), cancer mortality (p for interaction = 0.006), and CVD prevalence and its mortality (p for interaction = 0.003, p for interaction = 0.005, respectively). Furthermore, age emerged as a notable moderator in the relationship between dietary OBS and CVD prevalence, especially in individuals aged less than 45 years. Conversely, the protective effect of against cancer mortality differed from that for CVD, being more pronounced in individuals aged between 45 and 60 years (p for interaction = 0.017 and 0.006, respectively). However, there was a lack of significant interaction between these two factors and disease in lifestyle OBS (Supplementary Tables S4, S5).

### 3.6 Sensitivity analysis

In sensitivity analyses, we similarly observed that higher diet/lifestyle/overall OBS were associated with lower CVD prevalence in the USFLI-NAFLD cohort, and all exhibited a dose–response relationship, suggesting the stability of our findings (Supplementary Table S6). However, as USFLI-NAFLD relies on fasting samples to establish a diagnosis (and only a small proportion of NHANES participants currently have fasting biochemical testing), we lacked sufficient patient samples to conduct sensitivity analyses of the effect of OBS on mortality in NAFLD. Future studies are needed to incorporate larger samples for this validation.

## 4 Discussion

Our investigation encompassed 8,670 eligible participants diagnosed with NAFLD. Utilizing the comprehensive indicators of oxidative stress, specifically the OBS, we sought to elucidate how dietary and lifestyle factors, when adjusted within the practical context of prevalence and mortality of CVD in individuals with NAFLD.

The “Multiple hit” hypothesis currently stands as a fitting framework to elucidate the pathogenesis of NAFLD. Among various contributing factors, oxidative stress emerges as a primary instigator of injury and disease progression in NAFLD (24). Elevated generation of reactive oxygen species (ROS), in particular, exerts influence on hepatic lipid metabolism reprogramming, alterations in insulin sensitivity, and modulation of inflammation through interaction with innate immune signaling (25). Intriguingly, NAFLD and CVD share common risk factors, such as obesity, insulin resistance, and type 2 diabetes mellitus. Several studies have identified shared pathophysiological pathways between the two, with oxidative stress serving as a pivotal link. In the context of NAFLD, characterized by chronic inflammation, mitochondrial dysfunction, endoplasmic reticulum stress, and disrupted lipid metabolism are probable, collectively contributing to heightened cellular oxidative stress. This, in turn, triggers systemic inflammation, thrombosis, hypertension, and endothelial dysfunction, thereby instigating and propelling the onset and progression of CVD (26–29). Furthermore, clinical observation indicate that incorporating dietary antioxidants (including vitamin E, vitamin C, caffeine, coffee polyphenols, and hesperetin), along with lifestyle interventions like regular exercise, may enhance the prognosis of NAFLD in individuals with a heightened cardiometabolic risk, exerting beneficial effects through the modulation of oxidative stress (30). This is consistent with the results of our study. Our findings show that higher OBS quartiles show consistent associations with lower CVD prevalence across cardiovascular subtypes, underscoring the potential protective effects of an antioxidant rich diet and lifestyle choices in individuals with NAFLD. Additionally, we observed a linear relationship between overall OBS and the prevalence of CVD in NAFLD patients, underscoring the role of oxidative stress in the pathogenesis of CVD and emphasizing the importance of antioxidant interventions to mitigate oxidative damage. Furthermore, our observations suggest a potential does-response relationship, signifying that greater adherence to an antioxidative diet and lifestyle is associated with more substantial reductions in CVD prevalence within this population.

An interesting finding emerged from our investigation into the relationship between specific types of CVD and OBS: while lifestyle OBS exhibited an association with reduced risks of CHF and heart attack, dietary or overall OBS did not demonstrate such associations. Several factors may ecaudate this phenomenon. Firstly, known risk behaviors such as smoking and excessive alcohol intake contribute to the development of CHF and heart attack. Secondly, physical activity can positively impact heart function, blood pressure, and improve cholesterol levels (31). Furthermore, research indicates a potential dose–response relationship between elevated BMI and the risk of heart failure (32, 33). These factors, individually or synergistically, may directly influence cardiac health. Due to the necessity for efficient utilization of ingested nutrients by the body, influenced by individual variations in bioavailability and metabolism, a study assessing the bioavailability of iron, zinc, protein, and vitamin A in each diet of French adults revealed significant variability across individual dietary patterns (34). Consequently, in contrast to lifestyle factors, the impact of independent dietary OBS on specific types of CVD may manifest as more indirect and intricate. Secondly, CHF and heart attack often result from long-term CVD progression, whereas dietary habits may primarily affect heart health at a preventive or early stages. For instance, studies suggest that modest daily vitamin D and calcium supplementation may prevent CHF in postmenopausal women without prior CVD. However, such supplements were found to have no discernible effect in postmenopausal women with existing CVD (35). Although these supplements are also considered antioxidants within dietary OBS. In addition, psychosocial factors may also contribute to these outcomes (36).

While our findings align with prior research indicating the protective impact of antioxidants on NAFLD/CVD, the longitudinal correlation of OBS utilization with all-cause and CVD mortality has yet to be comprehensive investigated and substantiated in individuals with NAFLD. At present, most of the literature review is a diet or lifestyle impact on CVD/independent of NAFLD. For instance, the American Heart Association has introduced the concept of ideal cardiovascular health, encompassing seven metrics that require monitoring over time (37). Four lifestyle behaviors (BMI, smoking, diet, and physical activity) have demonstrated an inverse association with CVD and total mortality across diverse populations, including Chinese, Spanish, and other countries, and multi-ethnic groups (38, 39). Notably, recent study has revealed that leisure and transport-related physical activity (≥150 min/week) exerts a protective effect on all-cause and CVD mortality in patients with NAFLD (40). Physical activity constitutes a component of an antioxidant lifestyle. However, the impact of a singular factor on the entire oxidation/antioxidant system is limited, and OBS provides a comprehensively assessment of an individual’s overall REDOX status by capturing diverse dietary and lifestyle factors (41). Recent studies have further demonstrated the correlation between OBS and various disease, indicating that higher OBS levels can decrease periodontitis and reduce the risk of colorectal cancer, and it can improve the symptoms of sleep disorder and depression (42–45). In our study, OBS was considered both as continuous and categorical variables adjusting for all relevant confounders. In fully adjusted models, each incremental unit in diet OBS, lifestyle OBS, and overall OBS demonstrated a 2, 7, and 2% decrease in all-cause mortality, respectively, among NAFLD patients. This indicates a correlation between higher OBS and reduced all-cause mortality. Diet and overall OBS exhibited associations with decreased CVD mortality, suggesting their potential significance and positive impact on preventing CVD-related death in NAFLD patients. Furthermore, RCS analysis supported a linear relationship, showing that all OBS were linearly associated with CVD and all-cause mortality in NAFLD patients overall. However, overall OBS showed a nonlinear association with all-cause mortality, possibly suggesting a saturation point for behaviors combining an antioxidant diet and lifestyle, beyond which additional protective effects may not accrue. Additionally, different analytical methods may demonstrate varying sensitivities to different data patterns and trends. These findings underscore the need for more individualized consideration in developing prevention and intervention strategies for NAFLD patients, with future studies warranted to delve deeper into related mechanisms. In conclusion, in the absence of approved pharmacologic treatments for NAFLD, diet and lifestyle remain pivotal, and antioxidant factors show potential protective effects in this context (46).

In addition, our study explored cancer mortality in NAFLD, extrahepatic malignant tumors and HCC as prevalent causes of death in NAFLD patients, alongside CVD (47). Our findings revealed that lifestyle Q2 and Q3 were linked to reduced cancer mortality, whereas diet and overall OBS did not exhibit an association. Notably, lifestyle OBS demonstrated a nonlinear associated with cancer mortality. A prior NHAENS-based study indicated that high diet quality was associated with reduced levels in non-NAFLD population, but this association was not observed in NAFLD patients. Diet quality was assessed using the Healthy Eating Index (HEI) score (48). This contrasts with numerous previous studies highlighting the beneficial effects of diet and lifestyle on cancer-related mortality in individuals without NAFLD (49). However, cancer mortality in individuals with NAFLD may be influenced by specific lifestyle factors, and this association might vary based on cancer type. Studies have shown that aerobic exercise is negatively correlated with breast cancer and colorectal cancer mortality, but the mortality associated with liver cancer is more common in patients with NAFLD (50, 51).

Remarkably, in our stratified analyses, age (< 45 years) and education level (> high school) emerged as significant factors influencing association between OBS and CVD prevalence. Educational level also exerted a significant impact on the protective effect of OBS against all-cause and CVD mortality. There exists statistical evidence suggesting that as individuals reach the middle age of their biological lifespan, various pathological consequences start interfering with the cardiovascular system and metabolic processes. Notably, aging is intricately linked to oxidative stress, aligning with the “Free Radical Theory of Aging” proposed by D. Harman in the mid-1950s, highlighting increased reactive ROS production as a key aging factor (52). Aging-related dysregulation of hepatic lipid metabolism is mediated by disruption in nutrient sensing, mitochondrial dysfunction, autophagy, and the presence of senescent cells, playing a pivotal role in NAFLD-HCC progression (53). We hypothesize that younger individuals benefit more from an antioxidant diet due to their faster metabolic rate, enhanced digestion, and absorption capacity, which facilitates better management of the ROS generated during metabolism. Conversely, older adults exhibit slower metabolism, compromised liver function, reduced intestinal absorption, and immunosenescence (54). Furthermore, reliance on self-reported dietary data in our study presents unique challenges, particularly among older individuals. Cognitive decline and memory impairment in this demographic may have influenced responses to dietary questionnaires, potentially contributing to variations in outcomes (55). Nevertheless, initiating an antioxidant-rich diet and lifestyle from a younger age remains paramount for the prevention and management of NAFLD. Intriguingly, unlike CVD, the modifying effect of age on cancer mortality was more pronounced at the age of ≥45 years and < 60 years in dietary OBS. Firstly, as individuals age, notable changes occur in the body’s endocrine and immune systems. Moreover, different types of cancer exhibit variable incidence and mortality across age groups. For instance, an analysis of surveillance data on cancer mortality in China from 2005 to 2020 indicated that the top three fatal cancer among men and women aged 40–59 years were liver, tracheal, and bronchial cancers, whereas leukemia was the predominant cause of cancer-related death in the 0–19 year age group (56). This aligns with our findings, given that liver cancer is one of the leading causes of death in the NAFLD population (47). Thus, our results underscore the complexity of how various diseases respond to age and dietary influences. In addition, our findings consistently demonstrated that for both dietary and lifestyle OBS, possessing an education level exceeding high school was invariably linked with a reduced risk of diseases and mortality. A study indicated that each 4.2-year increase in education causally predicted a 52% reduction in NAFLD prevalence. Higher education levels correlate with increased awareness of the link between a healthy diet, physical activity, and reduced risk of chronic disease. This heighted awareness promotes the acquisition of health knowledge and the ability to adhere to health behavior, indirectly reducing morbidity and mortality. Education level, a crucial determinant of socioeconomic status, profoundly influences the prevalence of chronic diseases, often shaping an individual’s career tracery and income prospects (57). Given the considerable global disparities in education (58), addressing educational inequality holds significant implications, particularly in light of the protective role of education beyond high school as identified in this study among the NAFLD population. Enhanced educational opportunities not only foster broader health literacy but also empower individuals to adopt healthier behaviors and lifestyles. Consequently, this holistic approach can mitigate the risk of NAFLD and CVD, optimize healthcare resources allocation, and alleviate the burden on healthcare systems. Therefore, tackling educational disparities stands as a pivotal stride toward enhancing health management and advancing public health on a broader scale (59). However, we noted the lack of a significant interaction between these two factors and disease in lifestyle OBS. This may be because lifestyle factors themselves have a direct and strong impact on the risk of disease, an effect that remains relatively stable across different ages and educational level. Moreover, the characteristics of the sample and limitations of statistical methods might also affect the results.

In fact, several healthy dietary approaches, including the Dietary Approaches to Prevent Hypertension (DASH), Dietary inflammation Index (DII), high-protein diet, intermittent calorie restriction diet, and Mediterranean diet (MED), have demonstrated associations with a reduced risk of NAFLD/CVD (60–62). It is crucial to emphasize that these dietary patterns are not uniform, and specific dietary recommendations may vary based on individual conditions and specific health goals. Beyond dietary patterns, other factors such as regular physical exercise, maintaining a healthy weight, and smoking cessation equally played an equally important role. However, our research findings not only underscore the pronounced oxidative characteristics of adopting healthy dietary and lifestyle habits in NAFLD but also highlight the associated of prevalence and mortality of CVD in NAFLD patients. In the realm of oxidative stress, independent or combined adherence to healthful diet and lifestyle continues to exhibit a significant capacity to diminish the risk of disease and its progression.

This study boasts several notable strengths. Utilizing data from the nationally representative NHAENS database spanning from 1999 to 2018, the inclusion of large samples and long-term tracking enhance the study’s external validity, offering relatively comprehensive and common samples. Large-scale data facilitate the identification of differences across various subpopulations and yield more robust statistical outcomes. The incorporation of diverse statistical methods enriches study’s comprehensiveness, allowing for a multifaceted exploration of associations. However, certain limitations warrant acknowledgment. Firstly, the reliance on self-reported data from respondents introduces the potential for information bias, especially in the assessment of antioxidant diet and lifestyle. This may lead to uncertainty the results. Additionally, the long-term follow-up may entail missing data, potentially introducing population selection bias into the database. Secondly, the definition NAFLD using FLI, based on biochemical markers rather than liver biopsy, may lead to misclassification and an insufficient sample size to exclude related causes, including iron overload, genetic factors, and autoimmune hepatitis, thereby affecting diagnostic accuracy. Concurrently, the fasting glucose and insulin tests, essential for the USFLI-based NAFLD diagnosis, were conducted in only a limited subset of participants. Consequently, this resulted in an insufficient sample size to robustly conduct a sensitivity analysis assessing the impact of OBS on NAFLD mortality. In addition, despite utilizing multiple regression analysis to adjust for some confounding factors, there may still be unaccounted variables influencing the accuracy of the results. Given the observational design of the study, establishing causality is challenging. Potential confounders and reverse causality could affect the interpretation of study conclusions. Lastly, considering that the NHAENS database is derived from a US cohort, the extrapolation of our findings to different races and countries warrants additional investigation in future research endeavors.

All in all, persisting with a diet and lifestyle rich in antioxidant can significantly reduce all-cause mortality and prevalence and mortality of CVD in the NAFLD population in the United States, offering potential benefits to individuals with both NAFLD and CVD. In this study, OBS played an important role, with both diet and life OBS demonstrating independent or combined protective effects against disease prevalence rate and mortality, especially among younger individuals and those with higher levels of education. We strongly advocate for the individualized management and treatment of NAFLD patients. Additionally, we recommend that future research adopts more rigorous experimental designs and utilizes comprehensive data sources for further validation and the expansion of these findings.

## Data availability statement

Publicly available datasets were analyzed in this study. This data can be found at: https://www.cdc.gov/nchs/nhanes/index.htm.

## Ethics statement

The studies involving humans were approved by the National Center for Health Statistics (NCHS) and Research Ethics Review Board. The studies were conducted in accordance with the local legislation and institutional requirements. The participants provided their written informed consent to participate in this study.

## Author contributions

YinL: Conceptualization, Data curation, Formal analysis, Investigation, Methodology, Software, Writing – original draft. YipL: Project administration, Supervision, Writing – review & editing.
