# Supplementary material for: Adherence to an antioxidant diet and lifestyle is associated with reduced risk of cardiovascular disease and mortality among adults with nonalcoholic fatty liver disease: evidence from NHANES 1999–2018
Source: Front Nutr. 2024 Apr 8;11:1361567. doi: 10.3389/fnut.2024.1361567 (PMC11033446; doi:10.3389/fnut.2024.1361567)
Supplement: Supplementary file 1 [file Table_1.DOCX]

**Supplementary Table S1**. OBS allocation scheme.

| ­­­­­ | Male | | | Female | | | Property |
| --- | --- | --- | --- | --- | --- | --- | --- |
|  | 0 | 1 | 2 | 0 | 1 | 2 |  |
| **Dietary OBS components** |  |  |  |  |  |  |  |
| *Dietary fiber (g/d)* | <12.55 | 12.5-19.67 | >19.67 | <10.05 | 10.05-16.30 | >16.30 | A |
| *Carotene (RE/d)* | <98.62 | 98.62-305.85 | >305.85 | <98.06 | 98.06-383.92 | >383.92 | A |
| *Riboflavin (mg/d)* | <1.79 | 1.79-2.69 | >2.69 | <1.34 | 1.34-2.02 | >2.02 | A |
| *Niacin (mg/d)* | <20.64 | 20.64-29.75 | >29.75 | <14.51 | 14.51-21.85 | >21.85 | A |
| *Vitamin B6 (mg/d)* | <1.59 | 1.59-2.40 | >2.40 | <1.13 | 1.13-1.77 | >1.77 | A |
| *Total folate (mcg/d)* | <315.52 | 315.52-491.5 | >491.5 | <250.50 | 250.50-388.5 | >388.5 | A |
| *Vitamin B12 (mcg/d)* | <3.35 | 3.35-6.20 | >6.20 | <2.22 | 2.22-4.21 | >4.21 | A |
| *Vitamin C (mg/d)* | <42.40 | 42.40-113.15 | >113.15 | <38.00 | 38.00-98.40 | >98.40 | A |
| *Vitamin E (ATE) (mg/d)* | <5.82 | 5.82-9.41 | >9.41 | <4.53 | 4.53-7.52 | >7.52 | A |
| *Calcium (mg/d)* | <645.50 | 645.50-1072.50 | >1072.50 | <499.00 | 499-848.50 | >848.50 | A |
| *Magnesium (mg/d)* | <256.50 | 256.50-361.02 | >361.02 | <186.50 | 186.50-283.00 | >283.00 | A |
| *Zinc (mg/d)* | <9.74 | 9.74-15.10 | >15.10 | <6.73 | 6.73-10.74 | >10.74 | A |
| *Copper (mg/d)* | <1.12 | 1.12-1.57 | >1.57 | <0.85 | 0.85-1.28 | >1.28 | A |
| *Selenium (mcg/d)* | <94.90 | 94.90-141.75 | >141.75 | <67.75 | 67.75-99.5 | >99.5 | A |
| *Total fat (g/d)* | >107.44 | 69.8-107.44 | <69.80 | >75.81 | 50.94-75.81 | <50.94 | P |
| *Iron (mg/d)* | >19.17 | 12.88-19.17 | <12.88 | >14.32 | 9.65-14.32 | <9.65 | P |
| **Lifestyle OBS components** |  |  |  |  |  |  |  |
| *Physical activity (MET-minute/week)* | <415.68 | 415.68-1134.00 | >1134.00 | <264.13 | 264.13-843.27 | >843.27 | A |
| *Alcohol (drinks/d)* | >3 drinks/d | 2-3 drinks/d | ≤2 drinks/d | >2 drinks/d | 1-2 drinks/d | ≤1 drinks/d | P |
| *Body mass index (kg/m2)* | >29.17 | 25.54-29.17 | <25.54 | >28.64 | 23.74-28.64 | <23.74 | P |
| *Cotinine (ng/mL)* | >1.13 | 0.04-1.13 | <0.04 | >0.17 | 0.04-0.17 | <0.04 | P |

A stood for the antioxidant, P for the pro-oxidant, OBS, Oxidative balance score.

**Supplementary Table S2.** The relationship between OBS and specific CVD in the NAFLD population.

| **CORONARY HEART DISEASE** | Crude Model  OR (95%CI) P-value | Mode 1  OR (95%CI) P-value | Model 2  OR (95%CI) P-value |
| --- | --- | --- | --- |
| OBS.DIETARY | 0.97 (0.95, 0.99) 0.0010 | 0.98 (0.96, 1.00) 0.0220 | 0.98 (0.95, 1.01) 0.1811 |
| OBS.DIETARY |  |  |  |
| Q1 | Ref. | Ref. | Ref. |
| Q2 | 0.80 (0.60, 1.06) 0.1268 | 0.79 (0.57, 1.08) 0.1351 | 0.82 (0.58, 1.15) 0.2478 |
| Q3 | 0.61 (0.44, 0.84) 0.0033 | 0.63 (0.45, 0.88) 0.0073 | 0.67 (0.46, 0.99) 0.0447 |
| Q4 | 0.59 (0.43, 0.80) 0.0010 | 0.68 (0.48, 0.94) 0.0221 | 0.76 (0.49, 1.18) 0.2241 |
| P for trend | 0.0004 | 0.0154 | 0.1720 |
| OBS.LIFESTYLE | 0.98 (0.90, 1.06) 0.6075 | 0.82 (0.75, 0.89) <0.0001 | 0.83 (0.76, 0.90) <0.0001 |
| OBS.LIFESTYLE |  |  |  |
| Q1 | Ref. | Ref. | Ref. |
| Q2 | 0.94 (0.69, 1.30) 0.7258 | 0.70 (0.50, 0.98) 0.0420 | 0.71 (0.51, 1.00) 0.0506 |
| Q3 | 0.95 (0.70, 1.29) 0.7423 | 0.63 (0.45, 0.88) 0.0086 | 0.65 (0.46, 0.91) 0.0130 |
| Q4 | 0.91 (0.65, 1.28) 0.5992 | 0.48 (0.34, 0.69) 0.0001 | 0.50 (0.35, 0.71) 0.0002 |
| P for trend | 0.6148 | 0.0001 | 0.0002 |
| OBS | 0.97 (0.95, 0.99) 0.0014 | 0.97 (0.95, 0.99) 0.0036 | 0.97 (0.95, 1.00) 0.0319 |
| OBS |  |  |  |
| Q1 | Ref. | Ref. | Ref. |
| Q2 | 0.69 (0.54, 0.89) 0.0052 | 0.66 (0.50, 0.88) 0.0054 | 0.68 (0.49, 0.93) 0.0165 |
| Q3 | 0.54 (0.38, 0.76) 0.0006 | 0.53 (0.37, 0.76) 0.0008 | 0.55 (0.36, 0.82) 0.0045 |
| Q4 | 0.59 (0.43, 0.80) 0.0008 | 0.60 (0.43, 0.83) 0.0026 | 0.64 (0.42, 0.99) 0.0478 |
| P for trend | 0.0012 | 0.0047 | 0.0518 |
| **CONGESTIVE HEART FAILURE** |  |  |  |
| OBS.DIETARY | 0.94 (0.93, 0.96) <0.0001 | 0.96 (0.94, 0.99) 0.0022 | 0.98 (0.95, 1.01) 0.1887 |
| OBS.DIETARY |  |  |  |
| Q1 | Ref. | Ref. | Ref. |
| Q2 | 0.83 (0.58, 1.18) 0.2946 | 0.94 (0.64, 1.37) 0.7451 | 1.02 (0.68, 1.52) 0.9364 |
| Q3 | 0.56 (0.38, 0.81) 0.0027 | 0.70 (0.47, 1.04) 0.0807 | 0.79 (0.52, 1.20) 0.2714 |
| Q4 | 0.37 (0.25, 0.54) <0.0001 | 0.54 (0.35, 0.83) 0.0059 | 0.68 (0.37, 1.23) 0.2007 |
| P for trend | <0.0001 | 0.0018 | 0.1246 |
| OBS.LIFESTYLE | 0.89 (0.82, 0.97) 0.0083 | 0.79 (0.72, 0.87) <0.0001 | 0.80 (0.73, 0.88) <0.0001 |
| OBS.LIFESTYLE |  |  |  |
| Q1 | Ref. | Ref. | Ref. |
| Q2 | 1.43 (1.03, 1.98) 0.0350 | 1.24 (0.87, 1.75) 0.2380 | 1.29 (0.90, 1.86) 0.1623 |
| Q3 | 0.79 (0.53, 1.18) 0.2453 | 0.61 (0.40, 0.92) 0.0187 | 0.63 (0.42, 0.96) 0.0348 |
| Q4 | 0.70 (0.49, 1.01) 0.0611 | 0.48 (0.33, 0.71) 0.0003 | 0.51 (0.34, 0.75) 0.0009 |
| P for trend | 0.0032 | <0.0001 | <0.0001 |
| OBS | 0.95 (0.93, 0.96) <0.0001 | 0.96 (0.94, 0.98) 0.0002 | 0.97 (0.94, 1.00) 0.0255 |
| OBS |  |  |  |
| Q1 | Ref. | Ref. | Ref. |
| Q2 | 0.77 (0.54, 1.08) 0.1317 | 0.87 (0.60, 1.25) 0.4436 | 0.90 (0.62, 1.32) 0.6002 |
| Q3 | 0.44 (0.29, 0.67) 0.0001 | 0.55 (0.35, 0.86) 0.0092 | 0.59 (0.38, 0.93) 0.0234 |
| Q4 | 0.37 (0.25, 0.55) <0.0001 | 0.50 (0.33, 0.77) 0.0018 | 0.59 (0.33, 1.04) 0.0711 |
| P for trend | <0.0001 | 0.0005 | 0.0276 |
| **HEART ATTACK** |  |  |  |
| OBS.DIETARY | 0.97 (0.95, 0.99) 0.0025 | 0.99 (0.96, 1.01) 0.2054 | 0.98 (0.95, 1.01) 0.1205 |
| OBS.DIETARY |  |  |  |
| Q1 | Ref. | Ref. | Ref. |
| Q2 | 0.99 (0.71, 1.36) 0.9294 | 1.08 (0.76, 1.53) 0.6702 | 1.02 (0.70, 1.48) 0.9140 |
| Q3 | 0.69 (0.46, 1.02) 0.0638 | 0.81 (0.53, 1.22) 0.3087 | 0.74 (0.47, 1.16) 0.1896 |
| Q4 | 0.63 (0.45, 0.88) 0.0079 | 0.83 (0.57, 1.21) 0.3270 | 0.72 (0.44, 1.19) 0.2014 |
| P for trend | 0.0020 | 0.1797 | 0.1083 |
| OBS.LIFESTYLE | 0.95 (0.87, 1.04) 0.3131 | 0.84 (0.76, 0.93) 0.0009 | 0.85 (0.77, 0.94) 0.0017 |
| OBS.LIFESTYLE |  |  |  |
| Q1 | Ref. | Ref. | Ref. |
| Q2 | 1.48 (1.12, 1.95) 0.0060 | 1.26 (0.94, 1.68) 0.1206 | 1.28 (0.96, 1.71) 0.0972 |
| Q3 | 0.93 (0.65, 1.33) 0.6723 | 0.70 (0.48, 1.03) 0.0729 | 0.72 (0.49, 1.05) 0.0937 |
| Q4 | 0.94 (0.65, 1.34) 0.7241 | 0.61 (0.42, 0.89) 0.0122 | 0.62 (0.42, 0.92) 0.0174 |
| P for trend | 0.92 (0.83, 1.03) 0.1730 | 0.80 (0.71, 0.91) 0.0006 | 0.81 (0.72, 0.91) 0.0009 |
| OBS | 0.97 (0.95, 0.99) 0.0023 | 0.98 (0.96, 1.00) 0.0684 | 0.97 (0.94, 1.00) 0.0307 |
| OBS |  |  |  |
| Q1 | Ref. | Ref. | Ref. |
| Q2 | 0.88 (0.64, 1.19) 0.3970 | 0.95 (0.67, 1.34) 0.7574 | 0.89 (0.61, 1.29) 0.5367 |
| Q3 | 0.67 (0.44, 1.00) 0.0508 | 0.78 (0.51, 1.19) 0.2509 | 0.70 (0.44, 1.11) 0.1336 |
| Q4 | 0.63 (0.45, 0.89) 0.0106 | 0.77 (0.52, 1.13) 0.1846 | 0.66 (0.39, 1.11) 0.1206 |
| P for trend | 0.0066 | 0.1402 | 0.0867 |
| **STROKE** |  |  |  |
| OBS.DIETARY | 0.96 (0.94, 0.98) 0.0005 | 0.98 (0.96, 1.00) 0.0970 | 0.97 (0.95, 1.00) 0.0980 |
| OBS.DIETARY |  |  |  |
| Q1 | Ref. | Ref. | Ref. |
| Q2 | 0.83 (0.55, 1.25) 0.3640 | 0.92 (0.60, 1.40) 0.6856 | 0.89 (0.56, 1.42) 0.6273 |
| Q3 | 0.65 (0.44, 0.95) 0.0288 | 0.77 (0.51, 1.14) 0.1929 | 0.72 (0.46, 1.15) 0.1712 |
| Q4 | 0.54 (0.37, 0.79) 0.0017 | 0.75 (0.51, 1.11) 0.1553 | 0.70 (0.43, 1.16) 0.1665 |
| P for trend | 0.0008 | 0.1130 | 0.1146 |
| OBS.LIFESTYLE | 0.99 (0.89, 1.09) 0.7676 | 0.94 (0.85, 1.04) 0.2359 | 0.95 (0.85, 1.05) 0.3031 |
| OBS.LIFESTYLE |  |  |  |
| Q1 | Ref. | Ref. | Ref. |
| Q2 | 1.33 (0.94, 1.89) 0.1085 | 1.25 (0.88, 1.77) 0.2238 | 1.25 (0.88, 1.78) 0.2123 |
| Q3 | 0.91 (0.58, 1.42) 0.6676 | 0.79 (0.50, 1.26) 0.3333 | 0.82 (0.51, 1.30) 0.3970 |
| Q4 | 1.05 (0.68, 1.62) 0.8212 | 0.89 (0.57, 1.39) 0.6213 | 0.91 (0.58, 1.43) 0.6935 |
| P for trend | 0.6517 | 0.2220 | 0.2764 |
| OBS | 0.97 (0.95, 0.99) 0.0007 | 0.98 (0.96, 1.00) 0.0694 | 0.97 (0.95, 1.00) 0.0807 |
| OBS |  |  |  |
| Q1 | Ref. | Ref. | Ref. |
| Q2 | 0.79 (0.55, 1.14) 0.2063 | 0.87 (0.60, 1.28) 0.4905 | 0.83 (0.54, 1.28) 0.3975 |
| Q3 | 0.56 (0.36, 0.89) 0.0150 | 0.68 (0.42, 1.09) 0.1094 | 0.63 (0.36, 1.10) 0.1090 |
| Q4 | 0.53 (0.36, 0.79) 0.0018 | 0.71 (0.47, 1.06) 0.0942 | 0.64 (0.38, 1.06) 0.0863 |
| P for trend | 0.0005 | 0.0516 | 0.0534 |
| **ANGINA** |  |  |  |
| OBS.DIETARY | 0.96 (0.94, 0.99) 0.0026 | 0.97 (0.95, 1.00) 0.0420 | 0.97 (0.94, 1.00) 0.0961 |
| OBS.DIETARY |  |  |  |
| Q1 | Ref. | Ref. | Ref. |
| Q2 | 0.67 (0.47, 0.97) 0.0333 | 0.70 (0.48, 1.02) 0.0672 | 0.70 (0.46, 1.06) 0.0977 |
| Q3 | 0.51 (0.33, 0.79) 0.0027 | 0.56 (0.36, 0.85) 0.0083 | 0.56 (0.34, 0.92) 0.0226 |
| Q4 | 0.52 (0.35, 0.78) 0.0021 | 0.65 (0.42, 0.99) 0.0488 | 0.68 (0.40, 1.15) 0.1531 |
| P for trend | 0.0020 | 0.0421 | 0.1216 |
| OBS.LIFESTYLE | 0.87 (0.78, 0.96) 0.0060 | 0.77 (0.69, 0.86) <0.0001 | 0.78 (0.70, 0.86) <0.0001 |
| OBS.LIFESTYLE |  |  |  |
| Q1 | Ref. | Ref. | Ref. |
| Q2 | 0.87 (0.60, 1.27) 0.4758 | 0.73 (0.49, 1.09) 0.1267 | 0.74 (0.49, 1.11) 0.1451 |
| Q3 | 0.70 (0.48, 1.03) 0.0732 | 0.54 (0.36, 0.80) 0.0028 | 0.56 (0.37, 0.83) 0.0051 |
| Q4 | 0.58 (0.38, 0.90) 0.0160 | 0.39 (0.25, 0.61) <0.0001 | 0.40 (0.26, 0.62) 0.0001 |
| P for trend | 0.0092 | <0.0001 | <0.0001 |
| OBS | 0.96 (0.93, 0.98) 0.0015 | 0.96 (0.94, 0.99) 0.0094 | 0.96 (0.93, 0.99) 0.0132 |
| OBS |  |  |  |
| Q1 | Ref. | Ref. | Ref. |
| Q2 | 0.61 (0.43, 0.87) 0.0063 | 0.63 (0.44, 0.91) 0.0137 | 0.61 (0.41, 0.91) 0.0167 |
| Q3 | 0.47 (0.30, 0.73) 0.0009 | 0.51 (0.32, 0.79) 0.0036 | 0.49 (0.29, 0.81) 0.0059 |
| Q4 | 0.47 (0.31, 0.73) 0.0008 | 0.54 (0.35, 0.84) 0.0070 | 0.52 (0.31, 0.88) 0.0167 |
| P for trend | 0.0012 | 0.0097 | 0.0193 |

Model1: age, sex, race, marital, education, PIR; Model2: Model1+diabetes+hypertension+energy intake. OBS, Oxidative balance score; NAFLD, Nonalcoholic fatty liver disease; CVD, cardiovascular disease; PIR, family income to poverty ratio; OR odds ratio; 95% CI, 95% confidence interval; Ref, Reference.

**Supplementary Table S3.** This table presents data on all-cause death, CVD-cause death, and cancer-cause death, along with the median and IQR of time in months.

| **All-cause death** | **n** | **N** |
| --- | --- | --- |
| alive | 7424(89.44) | 35258894 |
| death | 1246(10.56) | 4164376 |
| **CVD-cause death** |  |  |
| No | 8249(96.53) | 38053368 |
| Yes | 421(3.47) | 1369902 |
| **Cancer-cause death** |  |  |
| No | 8348(97.09) | 38274100 |
| Yes | 322(2.91) | 1149170 |
| **Time, month(median,IQR)** | 115.0 (62.2-168.0) | 115.0 (62.2-168.0) |

n, sample size; N, the number of Americans represented, accounting for the applied weighting methodology. CVD, cardiovascular disease; IQR, interquartile range.

**Supplementary Table S4**. Analysis of the association of dietary OBS with age and education factors.

| **Dietary OBS.** | **OR/HR (95% CI)** | ***p*** | ***p* for interaction** |
| --- | --- | --- | --- |
| **CVD** |  |  |  |
| **Age** |  |  | 0.017 |
| <45 | 0.887(0.839,0.938) | <0.0001 |  |
| >=45, <60 | 0.990(0.956,1.025) | 0.564 |  |
| >=60 | 0.994(0.971,1.018) | 0.635 |  |
| **Education** |  |  | 0.003 |
| <high school | 0.971(0.912,1.034) | 0.357 |  |
| high school | 1.017(0.990,1.045) | 0.216 |  |
| >high school | 0.951(0.926,0.976) | <0.001 |  |
| **ALL-death** |  |  | 0.328 |
| **Age** | 0.951(0.913,0.991) | 0.016 |  |
| <45 | 0.947(0.906,0.990) | 0.016 |  |
| >=45, <60 | 0.981(0.960,1.002) | 0.078 |  |
| >=60 |  |  |  |
| **Education** |  |  | < 0.001 |
| <high school | 0.992(0.939,1.049) | 0.781 |  |
| high school | 0.992(0.964,1.020) | 0.56 |  |
| >high school | 0.931(0.905,0.958) | <0.0001 |  |
| **CVD-death** |  |  |  |
| **Age** |  |  | 0.334 |
| <45 | 0.905(0.824, 0.994) | 0.038 |  |
| >=45, <60 | 0.930(0.824,1.050) | 0.241 |  |
| >=60 | 0.980(0.952,1.008) | 0.152 |  |
| **Education** |  |  | 0.005 |
| <high school | 0.996(0.911,1.088) | 0.925 |  |
| high school | 0.991(0.948,1.035) | 0.674 |  |
| >high school | 0.911(0.875, 0.948) | <0.0001 |  |
| **CANCER-death** |  |  |  |
| **Age** |  |  | 0.003 |
| <45 | 0.975(0.890,1.068) | 0.583 |  |
| >=45, <60 | 0.919(0.868, 0.973) | 0.004 |  |
| >=60 | 0.998(0.959,1.040) | 0.937 |  |
| **Education** |  |  | 0.006 |
| <high school | 1.026(0.960, 1.097) | 0.445 |  |
| high school | 0.977(0.939,1.016) | 0.248 |  |
| >high school | 0.964(0.931,0.999) | 0.045 |  |

CVD, cardiovascular disease; OR odds ratio; HR, hazard ratio; 95% CI, 95% confidence interval.

**Supplementary Table S5**. Analysis of the association of lifestyle OBS with age and education factors.

| **Lifestyle OBS.** | **OR/HR (95%CI)** | ***p*** | ***p* for interaction** |
| --- | --- | --- | --- |
| **CVD** |  |  |  |
| **Age** |  |  | 0.163 |
| <45 | 0.832(0.672,1.029) | 0.089 |  |
| >=45, <60 | 0.838(0.747,0.941) | 0.003 |  |
| >=60 | 0.895(0.823,0.972) | 0.009 |  |
| **Education** |  |  | 0.945 |
| <high school | 0.880(0.724,1.070) | 0.197 |  |
| high school | 0.846(0.762,0.940) | 0.002 |  |
| >high school | 0.830(0.763,0.903) | <0.0001 |  |
| **ALL-death** |  |  |  |
| **Age** |  |  | 0.695 |
| <45 | 0.816(0.647,1.030) | 0.086 |  |
| >=45, <60 | 0.770(0.662,0.895) | <0.001 |  |
| >=60 | 0.826(0.767,0.890) | <0.0001 |  |
| **Education** |  |  | 0.291 |
| <high school | 0.686(0.559,0.842) | <0.001 |  |
| high school | 0.786(0.703,0.878) | <0.0001 |  |
| >high school | 0.741(0.665,0.825) | <0.0001 |  |
| **CVD-death** |  |  |  |
| **Age** |  |  | 0.435 |
| <45 | 0.748(0.503, 1.112) | 0.15 |  |
| >=45, <60 | 0.750(0.565, 0.996) | 0.047 |  |
| >=60 | 0.875(0.781,0.979) | 0.02 |  |
| **Education** |  |  | 0.443 |
| <high school | 0.810(0.570,1.152) | 0.237 |  |
| high school | 0.810(0.697,0.941) | 0.006 |  |
| >high school | 0.747(0.643, 0.867) | <0.001 |  |
| **CANCER-death** |  |  |  |
| **Age** |  |  | 0.114 |
| <45 | 0.677(0.449,1.021) | 0.063 |  |
| >=45, <60 | 0.782(0.652,0.939) | 0.009 |  |
| >=60 | 0.884(0.799,0.979) | 0.017 |  |
| **Education** |  |  | 0.681 |
| <high school | 0.805(0.600, 1.080) | 0.145 |  |
| high school | 0.837(0.694,1.009) | 0.062 |  |
| >high school | 0.790(0.644,0.968) | 0.024 |  |

CVD, cardiovascular disease; OR odds ratio; HR, hazard ratio; 95% CI, 95% confidence interval.

**Supplementary Table S6.** The associations between diet/lifestyle/overall OBS and the prevalence of CVD within the USFLI-NAFLD cohort.

| **Outcome: CVD** | Crude Model  OR (95%CI) P-value | Model 1  OR (95%CI) P-value | Model 2  OR (95%CI) P-value |
| --- | --- | --- | --- |
| OBS.DIETARY | 0.97 (0.95, 0.98) <0.0001 | 0.98 (0.97, 1.00) 0.0472 | 0.98 (0.97, 1.00) 0.0525 |
| OBS.DIETARY |  |  |  |
| Q1 | Ref. | Ref. | Ref. |
| Q2 | 0.78 (0.60, 1.02) 0.0670 | 0.85 (0.64, 1.13) 0.2619 | 0.86 (0.65, 1.14) 0.2989 |
| Q3 | 0.65 (0.50, 0.84) 0.0012 | 0.77 (0.58, 1.03) 0.0772 | 0.78 (0.58, 1.04) 0.0912 |
| Q4 | 0.54 (0.42, 0.71) <0.0001 | 0.73 (0.55, 0.99) 0.0396 | 0.73 (0.55, 0.99) 0.0422 |
| P for trend | <0.0001 | 0.0293 | 0.0318 |
| OBS.LIFESTYLE | 0.99 (0.92, 1.07) 0.8143 | 0.91 (0.84, 0.99) 0.0261 | 0.91 (0.84, 0.98) 0.0183 |
| OBS.LIFESTYLE |  |  |  |
| Q1 | Ref. | Ref. | Ref. |
| Q2 | 1.20 (0.89, 1.62) 0.2362 | 1.17 (0.84, 1.61) 0.3497 | 1.17 (0.84, 1.62) 0.3462 |
| Q3 | 0.93 (0.68, 1.26) 0.6294 | 0.78 (0.56, 1.10) 0.1560 | 0.79 (0.56, 1.10) 0.1650 |
| Q4 | 0.98 (0.73, 1.31) 0.8817 | 0.77 (0.56, 1.06) 0.1121 | 0.75 (0.54, 1.04) 0.0843 |
| P for trend | 0.3344 | 0.0084 | 0.0052 |
| OBS | 0.97 (0.96, 0.98) <0.0001 | 0.98 (0.97, 1.00) 0.0188 | 0.98 (0.97, 1.00) 0.0198 |
| OBS |  |  |  |
| Q1 | Ref. | Ref. | Ref. |
| Q2 | 0.70 (0.54, 0.91) 0.0072 | 0.74 (0.56, 0.98) 0.0372 | 0.73 (0.55, 0.97) 0.0317 |
| Q3 | 0.59 (0.45, 0.78) 0.0002 | 0.71 (0.52, 0.95) 0.0223 | 0.71 (0.53, 0.96) 0.0282 |
| Q4 | 0.54 (0.41, 0.71) <0.0001 | 0.69 (0.51, 0.93) 0.0162 | 0.68 (0.50, 0.93) 0.0144 |
| P for trend | <0.0001 | 0.0185 | 0.0190 |

Model1: age, sex, race, marital, education, PIR; Model2: Model1+diabetes+hypertension+energy intake. OBS, Oxidative balance score; NAFLD, Nonalcoholic fatty liver disease; CVD, cardiovascular disease; PIR, family income to poverty ratio; OR odds ratio; 95% CI, 95% confidence interval; ref, reference.

**
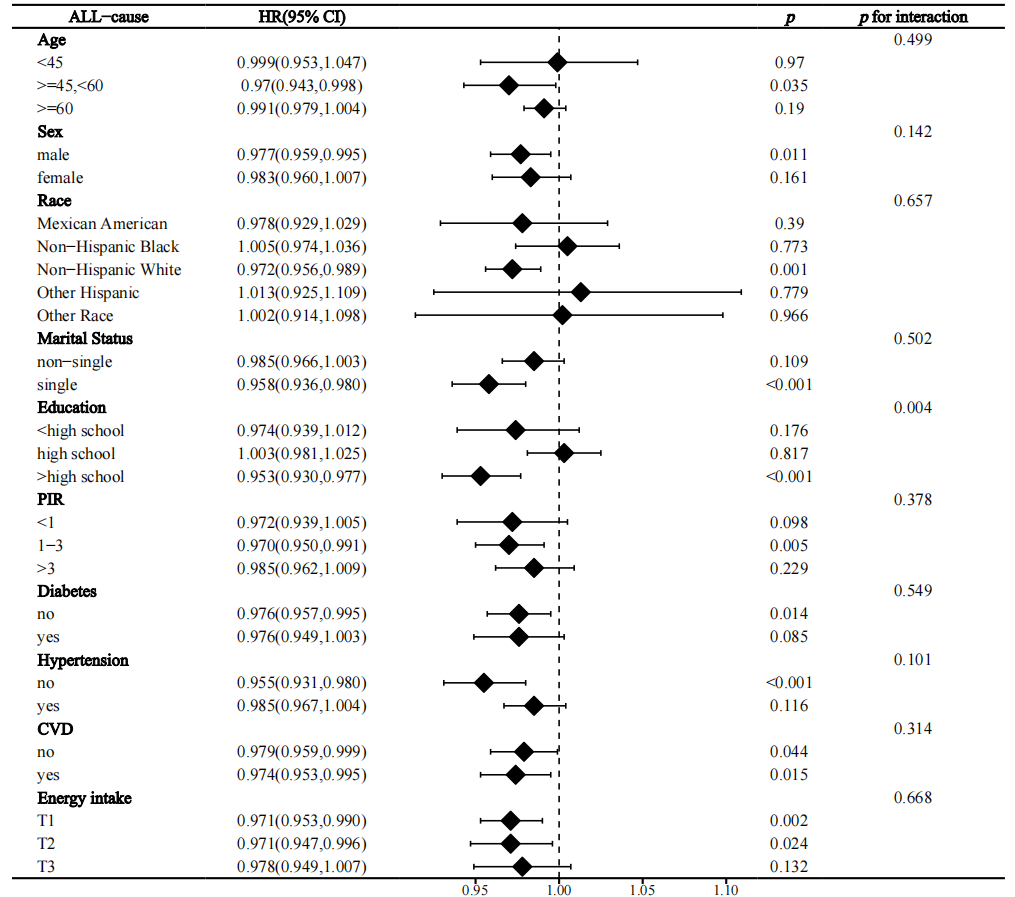
**

**Supplementary Figure 1.** Forest plot the relationship between OBS and the prevalence of all- cause mortality in NAFLD. OBS, Oxidative balance score; NAFLD, Nonalcoholic fatty liver disease; CVD, cardiovascular disease; PIR, family income to poverty ratio; HR, hazard ratio; 95% CI, 95%confidence interval.

**
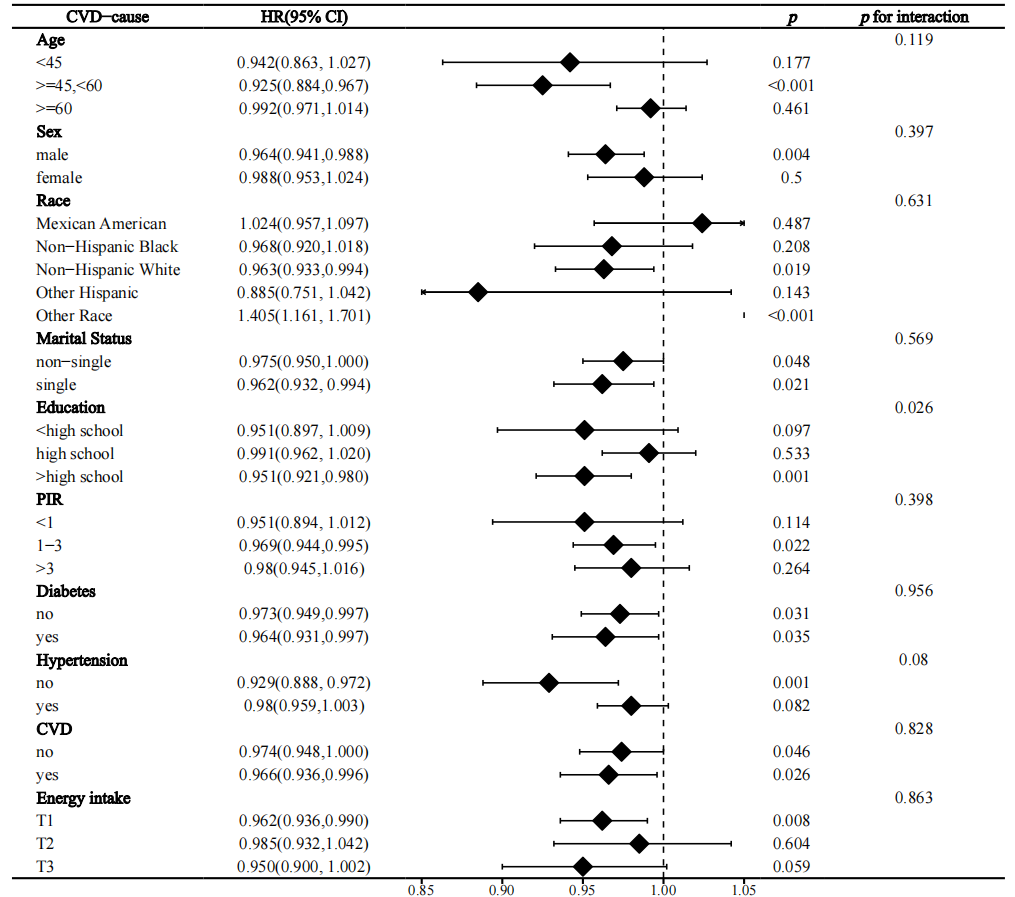
**

**Supplementary Figure 2.** Forest plot the relationship between OBS and the prevalence of CVD- cause mortality in NAFLD. OBS, Oxidative balance score; NAFLD, Nonalcoholic fatty liver disease; CVD, cardiovascular disease; PIR, family income to poverty ratio; HR, hazard ratio; 95% CI, 95%confidence interval.

**
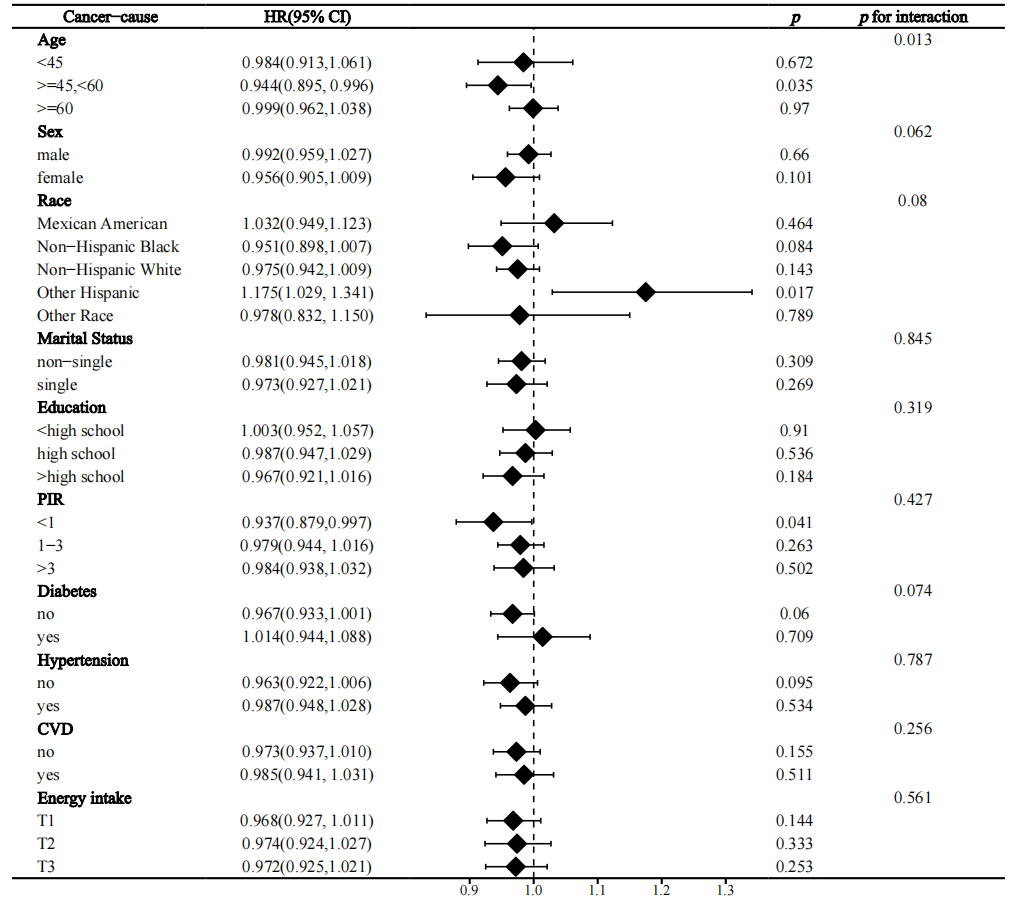
**

**Supplementary Figure 3.** Forest plot the relationship between OBS and the prevalence of cancer- cause mortality in NAFLD. OBS, Oxidative balance score; NAFLD, Nonalcoholic fatty liver disease; CVD, cardiovascular disease; PIR, family income to poverty ratio; HR, hazard ratio; 95% CI, 95% confidence interval.
